# Valorization of Baltic Sea farmed blue mussels: Chemical profiling and prebiotic potential for nutraceutical and functional food development

**DOI:** 10.1016/j.fochx.2024.101736

**Published:** 2024-08-13

**Authors:** Indrek Adler, Jonne Kotta, Marju Robal, Sanjida Humayun, Kristel Vene, Rando Tuvikene

**Affiliations:** a Estonian Maritime Academy, Tallinn University of Technology, Kopli 101, 11712 Tallinn, Estonia; b Estonian Marine Institute, University of Tartu, Mäealuse 14, 12618 Tallinn, Estonia; c School of Natural Sciences and Health, Tallinn University, Narva mnt 25, 10120 Tallinn, Estonia; d Tallinn University of Technology, School of Science, Department of Chemistry and Biotechnology, Akadeemia tee 15, 12618 Tallinn, Estonia

**Keywords:** Mytilus, Valorization, Amino acids, Fatty acids, Glycogen, Prebiotics

## Abstract

The severe eutrophication of the Baltic Sea requires mussel (*Mytilus* spp.) farming to remove nutrients, but farming in a low salinity environment results in smaller mussels that require value enhancement to be economically viable. This study evaluates the biomass valorisation of smaller Baltic mussels, focusing on the extraction of oil, protein and glycogen. It analyses the amino acid profiles, oil and fatty acid contents and glycogen levels of the mussels, as well as their prebiotic properties on beneficial gut bacteria. In addition, the study improves the extraction of bioactive compounds through enzymatic hydrolysis. Results indicate significant seasonal differences, with summer mussels having higher meat and lower ash content, and a rich content of essential fatty acids, particularly omega-3, and amino acids, underscoring the mussels' sustainability as a food source. The enzymatically treated biomass exhibited notable prebiotic activity, proposing health-promoting benefits. The study underscores the valorization of Baltic mussel biomass, highlighting its role in health, nutrition, and environmental sustainability.

## Highlights

• Baltic Sea farmed mussels exhibit strong seasonal variability in their chemical profile and prebiotic potential.
• Summer-harvested mussels have the highest content of meat, essential fatty acids, and amino acids.
• Enzymatic hydrolysis with subtilisin enhances the extraction of bioactive compounds.
• Enzyme-treated mussel biomass shows significant prebiotic activity.

## 1 Introduction

Marine ecosystems boast a vast diversity of species, many of which are currently used as sources of protein and industrial raw materials. However, <10% of marine bioresources are utilized for human food and other purposes (Pauly, 2007). The exploration of marine bioresources as sources of functional food, feed, cosmetics, pharmaceuticals, and biomedical research is on the rise (Rotter et al., 2021).

Mussels are no exception; they are not only valuable for human consumption but also serve as a promising source of essential nutrients for shrimp and possess excellent chemo-attractant properties for fish. Furthermore, mussels have been identified as commercially significant, new, and potential biomaterial resources. Their polysaccharides, enzymes, peptides, lipids, and biominerals have various applications in the biomedical field, including hard and soft tissue engineering, bio-adhesives, dental biomaterials, and drug and cell delivery systems (Eroldoğan et al., 2023; Rotter et al., 2021).

In the Baltic Sea, the cultivation of mussels, in particular *Mytilus edulis*, *Mytilus trossulus* and their hybrids, presents a unique combination of challenges and opportunities. Despite years of attempts to control nutrient inputs, the Baltic Sea continues to suffer from severe eutrophication. This persistent problem is attributed to the accumulation of legacy nutrients (Andersen et al., 2017). In this context, low trophic aquaculture is emerging as a promising solution to effectively remove these excess nutrients from the ecosystem (Kotta et al., 2020). However, due to the low salinity of the region, these bivalves are smaller than their oceanic counterparts and therefore require a thorough biomass valorization strategy for sustainable business viability (Adler et al., 2022; Petersen and Stybel, 2022). The smaller size of these mussels, while a challenge, also presents an opportunity to develop innovative processing methods that can fully exploit the entire biomass for food, feed, and other purposes, thereby contributing to the economic viability and ecological sustainability of mussel farming in this region (Kotta et al., 2020; Maar et al., 2023). However, this must be done alongside efforts to change human attitudes. Currently, mussels are relatively unpopular as a mass-market food compared to other meat items, limiting our ability to achieve their potential environmental and health benefits. Increased publicity that takes into account regional and cultural differences in attitudes, emphasizing the health and environmental benefits of mussel meat, is essential. Additionally, industry engagement to develop a diverse range of appealing, affordable, and convenient mussel products is crucial for driving growth in the bivalve sector in the Baltic Sea region and beyond (Gawel et al., 2023). Furthermore, economic incentives, such as government subsidies and grants for sustainable aquaculture, are likely to encourage Baltic farmers to adopt sustainable practices (Sokołowski et al., 2022).

Previous research has shown that these mussels have a diverse and rich nutrient profile in the Baltic Sea and beyond (Azpeitia et al., 2016; Kube et al., 2007; Oliveira et al., 2015). They are rich in essential fatty acids, high-quality proteins, and glycogen, which provide a significant nutritional advantage. Notably, they contain omega-3 fatty acids, including EPA and DHA, which are widely recognized for their health benefits, such as reducing inflammation and providing cardioprotective effects (Calder, 2015; Jeromson et al., 2015; Zanetti et al., 2015). Additionally, the protein content in these mussels includes all essential amino acids, making them an even more valuable source of sustainable and premium nutrition that is vital for human health (De Swaan & Wijsman, 1976; Wu, 2009; Şereflişan & Altun, 2018).

In addition to their historical use in various culinary traditions, mussels are now being explored as a sustainable source of nutraceuticals and functional foods. This shift goes beyond basic nutrition and highlights their potential in the nutraceutical industry (FAO, 2018; Granato et al., 2023). This tendency has been significantly influenced by technological advancements in bioprocessing, particularly enzymatic hydrolysis. Techniques such as the use of specific enzymes, like subtilisin, have been essential in improving the extraction and refinement of bioactive compounds from shellfish biomass. This process has led to the breakdown of proteins into bioactive peptides and amino acids. Mussel-derived bioactive components are recognized for their various health-promoting effects, such as antihypertensive, antioxidative, and antimicrobial properties (Naik et al., 2020). This expands the potential of mussel-derived products in health and nutrition (Gupta et al., 2002; Harnedy & FitzGerald, 2012; Jin et al., 2016; Kim & Wijesekara, 2010; Nag et al., 2022).

Intestinal microbiota, particularly *Lactobacillus* and *Bifidobacterium* species, enhance gut health by producing short-chain fatty acids (van den Berg et al., 2021) and inhibiting the growth of pathogenic bacteria such as *Escherichia*, *Shigella*, and *Streptococcus* species (Dominguez-Bello et al., 2019). Prebiotics, including various polysaccharides like inulin, remain undigested in the small intestine and promote gut health by stimulating the growth of probiotic microbes. They also suppress the growth of pathogenic bacteria through antagonistic activity and modulate both pro-inflammatory and anti-inflammatory responses (Saltzman et al., 2017). Extracts from various mussel species, including proteins, peptides, amino acids, lipids, and polysaccharides, have demonstrated antibacterial and prebiotic activities, as well as antioxidant, antihypertensive, and anticoagulant properties (Saltzman et al., 2017). Peptides may also influence the growth and diversity of beneficial gut microbes, functioning as prebiotics (Maury, 2018). The novel prebiotic effect of the mussel *Perna canaliculus* has been reported, potentially related to glucosamine or similar compounds (Coulson et al., 2013; Siriarchavatana, 2021). Similarly, cysteine-rich antimicrobial peptides isolated from the blue mussel have shown strong bactericidal activity against both Gram-positive and Gram-negative bacteria (Charlet et al., 1996). Thus, advances in this field not only improve the quality of extracts, but also open up new avenues for their use in various health-promoting products (Durazzo et al., 2022).

Based on Adler et al. (2022), the current study aims to delineate the chemical composition of Baltic blue mussel biomass, focusing on the extraction, characterization, and prebiotic potential evaluation of oils, proteins, and glycogen. It further assesses the prebiotic efficacy of these mussel-derived fractions against other marine biomass, such as fish and algae, noted for their bioactive properties (Holdt & Kraan, 2011; Lordan et al., 2011; Venugopal, 2011). This research integrates advanced biotechnological methods with traditional fractionation techniques to underscore the value of the Baltic blue mussel as a sustainable health industry ingredient, contributing to novel health-promoting product development. Through a comprehensive analysis, encompassing the extraction, characterization, and evaluation of various bioactive compounds from mussel biomass, the study aims to advance our understanding of mussel biomass valorization, potentially revolutionizing nutraceutical and functional food innovations and highlighting the sustainability and versatility of blue mussels as a significant resource in health-related industries.

When designing the study, we had the following expectations: (1) We anticipate significant seasonal differences in the meat, fatty acids, and amino acids of harvested mussels; (2) Enzymatic hydrolysis with subtilisin increases the extraction yield of essential amino acids from Baltic mussel biomass, thereby enhancing its prebiotic effects and nutritional value; (3) The valorization process of Baltic mussel biomass through specific biochemical techniques can significantly enhance its nutritional, health-promoting and commercial value.

## 2 Materials and methods

### 2.1 Origin of mussels

The mussel biomass for this study was sourced from a mussel farm located in Tagalaht (58.456° N, 22.055° E) in the Baltic Sea, harvested at depths ranging from 0 to 3 m. The mussels were collected on four occasions: 23 September 2020, 23 November 2021, 30 July 2022 and 26 October 2022. Mussels were not harvested during the spring months, as this season is known for their poor condition. This is evidenced by their protein content, which peaks during autumn and winter and declines in the spring (Wołowicz et al., 2006). The age of the mussels at the time of collection was 1–1.5 years. The collected biomass was wet packed in 1 L portions in plastic bags and stored at −80 °C until laboratory analysis.

### 2.2 Biomass processing and fractionation

#### 2.2.1 General processing schemes

**Fig. 1:**
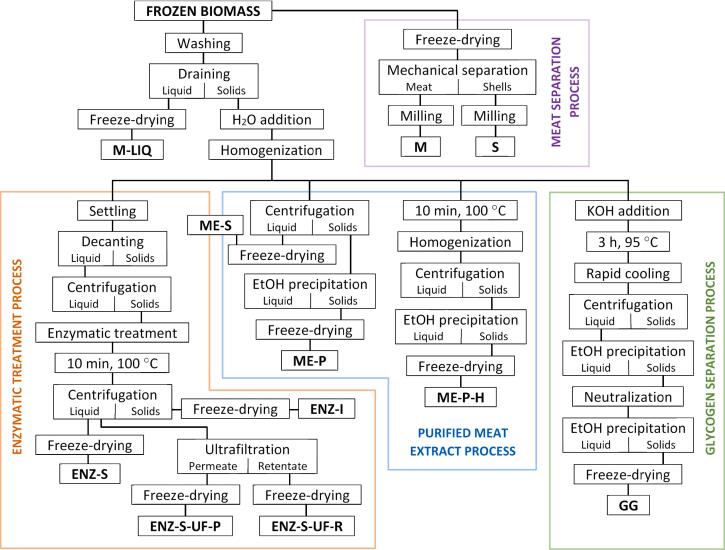
Schematic overview of the methods employed to derive diverse fractions from blue mussel biomass. (For interpretation of the references to colour in this figure legend, the reader is referred to the web version of this article.)

The methods used to obtain different fractions from the mussel biomass are illustrated in Fig. 1, while Table 1 shows the fractions obtained, each characterized by its unique chemical composition. The processes involved soaking the frozen biomass in equal mass of water until completely thawed, removing most of the liquid part by draining, resulting in the sample labeled ‘M-LIQ’ after freeze-drying. Subsequently, an equal mass of water was added to the solid part, and the mixture was homogenized using a Philips ProBlend 6 3D blender at maximum speed for 3 min. This yielded the homogenized mussel slurry, utilized in further steps, with the exception of the meat separation process.

**Table 1:** Fractions derived from processing the biomass of the blue mussel farm, with numbers following letters indicating the timing of biomass collection.

Abbreviation(s)	Description	Mussel collection time
M-1, M-2, M-3, M-4	Mechanically separated mussel meat	Sept 2020, Nov 2021, July 2022, Oct 2022
B-2, B-3	Mechanically separated barnacle meat	Nov 2021, July 2022
S-1, S-2, S-3, S-4	Mechanically separated mussel shells	Sept 2020, Nov 2021, July 2022, Oct 2022
M-LIQ	Substances from thawed water released from mussel biomass	July 2022
ME-S	Soluble part of homogenized mussel slurry	July 2022
ME-P	Purified mussel meat extract using a room-temperature process	July 2022
ME-P-H	Purified mussel meat extract using a high-temperature process	July 2022
GG	Glycogen-rich fraction from mussel biomass	July 2022
ENZ-S	Soluble part of subtilisin-treated mussel meat	July 2022
ENZ-I	Insoluble part of subtilisin-treated mussel meat	July 2022
ENZ-S-UF-P	Ultrafiltration permeate of soluble part of subtilisin-treated mussel meat	July 2022
ENZ-S-UF-R	Ultrafiltration retentate of soluble part of subtilisin-treated mussel meat	July 2022

#### 2.2.2 Meat separation process

Freeze-drying makes it easy to separate the mussel flesh from the shell, unlike high temperature drying, which makes it difficult to separate the flesh. During the meat separation process, barnacles, algae and empty shells were removed from the freeze-dried biomass to ensure a selection of pure mussels. Opened mussels were also excluded to avoid loss of dry matter from the meat during the freeze-drying process. After drying, approximately 70 mussels were carefully opened using a thin spatula and the dried flesh adhering to the shell was removed using the same instrument. Both the meat and the shell were then ground independently in a water-cooled laboratory mill (IKA A10 basic, Germany) to avoid heating the sample. This flesh separation technique was similarly applied to approximately 140 barnacles. This study included data on barnacles, as these organisms grow on mussel shells and inevitably become part of the mussel biomass to some extent, thereby affecting the chemical composition of the resulting products.

#### 2.2.3 Purified meat extract process

The purification of the mussel flesh fractions by the removal of less hydrophilic compounds and low molecular weight substances was achieved by an alcohol precipitation process. For this the homogenized mussel slurry was centrifuged at 12000*g* for 10 min at 4 °C to separate the supernatant (sample after freeze-drying as ‘ME-S') from the residue. An equal volume of 96% ethanol was added to the residue, mixed and kept for 24 h at 4 °C. This precipitate was then separated and freeze-dried to obtain fraction ‘ME-P'.

Similar procedure was employed to obtain the heat-treated purified meat extract. This was achieved by initially treating the homogenized mussel slurry for 10 min in a boiling water bath, followed by additional homogenization. The sequential centrifugation and ethanol precipitation steps were as previously described, resulting in the fraction ‘ME-P-H' after freeze-drying.

#### 2.2.4 Enzymatic treatment process

The homogenized mussel slurry was allowed to settle briefly, after which the upper flesh-rich part was decanted and the crushed shells were discarded. The decanted part was then centrifuged at 4500*g* for 5 min at 20 °C, the obtained precipitate was diluted with equal mass of water. The enzyme subtilisin (2.4 U/g) from *Bacillus licheniformis* (Sigma, P4860) was added to the diluted residue (pH = 7.9) to attain a final concentration of 1% in the mixture. The mixture was enzymatically treated for 2 h at 60 °C in a water bath equipped with a magnetic stirrer. Subsequently, the enzyme was inactivated by heating the solution in boiling water for 10 min. The inactivated and cooled sample was then centrifuged twice at 20 °C for 10 min at 12000*g*, resulting in separate collections of centrifuged residue and supernatant. The insoluble residue, representing to enzyme-resistant part of the sample, was subjected to freeze-drying, resulting in the sample named ‘ENZ-I'. Simultaneously, a portion of the supernatant containing solubilized proteins, peptides, and amino acids was also freeze-dried to produce the sample labeled ‘ENZ-S'. Another portion of the supernatant was fractionated by ultrafiltration technique using VivaFlow 200 PES membrane (Sartorius, Germany) with molecular weight cut-off of 10 kDa. The process involved reducing the sample volume by 15 times, followed by washing the retentate with water, using a volume equivalent to 13 times that of the original sample. This procedure produced two fractions: the low-molecular weight permeate (ENZ-S-UF-P) and the ultrafiltration retentate (ENZ-S-UF-R).

#### 2.2.5 Glycogen separation process

The homogenized mussel slurry was subjected to alkali treatment procedure by adding 100 g KOH per 500 g (∼800 mL) of the starting raw biomass. This mixture was then heated in a water bath at 95 °C for 30 min. After heating, the treated material was rapidly cooled to room temperature in a cold water bath. It was then centrifuged at 12000*g* for 10 min at 20 °C. After centrifugation, 1.5 times the volume of 96% ethanol was added to the supernatant. The precipitate obtained was separated by centrifugation. To reduce the alkaline residue, this ethanol precipitation step was repeated three times. A small amount of water was then added to the final precipitate, which was carefully neutralized with an aqueous 1 M HCl solution until an acidic reaction was achieved. Soluble biopolymers were then separated from the solution by adding ethanol again and centrifuging. This precipitation step was repeated to ensure a thorough separation. The final precipitated glycogen-rich fraction was freeze-dried, resulting in the production of sample named ‘GG’.

### 2.3 Dry matter and ash content determination

The dry matter and ash contents of the samples were determined gravimetrically. Freeze-drying was used to dry the samples. Prior to ashing, all samples were freeze-drying, then initially ashed in a muffle furnace at 550 °C for 6 h, followed by cooling in a desiccator, weighing and further ashing at 950 °C for 3 h.

### 2.4 Chemical analyses

#### 2.4.1 Protein and amino acid analysis

The amino acid content of freeze-dried and cryogenically ground mussel flesh or flesh extracts was determined by gas chromatography coupled to mass spectrometry (GC–MS). For the determination of total (proteinaceous and free) amino acids in biomass (including protein composition), 0.02 g of dry sample was hydrolyzed with 2 mL of 6 M HCl solution in hermetically sealed glass tubes at 120 °C for 15 h. The hydrolyzed sample was dried at 95 °C under a stream of nitrogen and dissolved in 2 mL of water. To determine the free amino acids, 0.05 g of the wet sample (mussel extract) was added to 1.5 mL of 0.1 M HCl and shaken vigorously at 1400 rpm for 5 min at room temperature. The mixture was then centrifuged at 4 °C for 15 min at 21000*g* and the supernatant was stored at −80 °C until analysis.

For gas chromatographic analysis, to 100 μL of the amino acid solution obtained in the previous steps, 250 μL of acetonitrile was added, shaken and centrifuged at 21000*g* for 3 min. 100 μL of the supernatant was pipetted into a heat-resistant, capped Eppendorf tube and 100 μL of internal standard solution (5 μg/mL DL-norleucine) was added. The sample was evaporated under a stream of nitrogen, 50 μL of dichloromethane was added, gently vortexed and evaporated again under a stream of nitrogen. To the dried sample, 100 μL MTBSTFA (Supelco, 77,626) and 100 μL acetonitrile were added and thoroughly mixed. The mixture was heated at 100 °C for 1 h, then centrifuged at 4 °C for 15 min at 21000*g* and transferred to a 200 μL sample vial. The vial was centrifuged again at 2000 rpm for 5 min and then analyzed by GC–MS. Amino acid concentrations were determined using a Shimadzu GCMS-QP2010 Ultra gas chromatograph system equipped with a mass detector (MS) and a Phenomenex Zebron ZB-5MS silicon-filled capillary column (30 m × 0.25 mm, 0.25 μm layer thickness). Helium was used as the carrier gas at a flow rate of 1 mL/min. The sample injector operated at 280 °C using a 2 mm diameter straight liner. The MS detector operated at 325 °C and the ion source at 300 °C. Scans ranged from *m*/*z* = 25–500, sample injection was in split mode (distribution flow 100) and the sample injection volume was 0.5 μL. In the analysis program the column was held at 100 °C for 2 min, then heated to 298 °C at 5 °C/min and held for 25 min. Quantification was performed using analytical standards (Supelco A6407, A6282).

#### 2.4.2 Lipid and fatty acid analysis

The lipid content was determined in freeze-dried and cryogenically ground samples of mussel flesh or flesh extracts using a chloroform:methanol (2:1, *v*/v) mixture for extraction. Approximately 0.3 g of homogenized sample was placed in a glass test tube to which 1 mL of methanol and 2 mL of chloroform were added. The suspension was shaken vigorously for 90 s and then incubated in an ultrasonic bath at 40 °C for 30 min. Then 1.25 mL of aqueous 2% NaCl solution and 1.25 mL of chloroform were added and shaken vigorously. The sample was centrifuged at 1700*g* for 20 min and the chloroform layer was transferred to a pre-weighed glass extraction tube using a glass Pasteur pipette. Organic solvents were removed by drying in a stream of nitrogen, and the remaining mass of the sample (mussel oil) was expressed as the percentage of lipids relative to the original freeze-dried material.

Fatty acid methyl esters (FAME) were prepared, and their content quantified by gas chromatography. Approximately 0.05 g of the previously obtained mussel oil was weighed into a glass test tube and 1.5 mL of 5% sulphuric acid solution in methanol was added. The mixture was heated in a water bath at 50 °C for 1 h, shaking gently for 30 s every 15 min. The tubes were then cooled in a mixture of ice and water, and 1 mL of water and 1.5 mL of hexane were added to the samples. The mixture was shaken vigorously and allowed to stratify. The top layer was transferred to a new glass tube using a glass Pasteur pipette, dried under a nitrogen stream and 500 μL of hexane was added. Samples were stored at −20 °C until GC analysis.

The fatty acid methyl esters obtained in the previous step were quantified using a Shimadzu GCMS-QP2010 Ultra gas chromatograph system with a mass spectrometric detector and a Phenomenex Zebron ZB-5MS silica-filled capillary column (30 m × 0.25 mm, 0.25 μm layer thickness). Helium was used as the carrier gas at a flow rate of 30 cm/s. The injection apparatus operated at 280 °C. The MS detector operated at 325 °C and the ion source at 300 °C. The scan range was *m*/*z* = 25–500 and the sample was injected in split mode (split flow 100) with a sample volume of 1 μL. In the analysis program the column temperature was raised from 160 °C to 260 °C at 2.5 °C/min, then to 298 °C at 5 °C/min and held for 15 min. The results were expressed as mass percentage of the total fatty acids identified. The standard mixtures PUFA-2 (Sigma, 47,015 U) and 38 FAME Mix (Supelco CRM47885) were used for fatty acid identification/quantification.

#### 2.4.3 Glycogen content analysis

Glycogen content was determined in freeze-dried and cryogenically ground (Retsch cryomill, Germany) samples of mussel flesh or flesh extracts using alkaline extraction and spectrophotometric detection with phenol-sulphuric acid reagent. In order to prepare samples, 0.001–0.005 g of freeze-dried mussel flesh was placed in screw-capped Eppendorf tubes to which 100 μL of 30% KOH aqueous solution was added. The mixture was carefully stirred and heated in a thermoshaker at 99 °C for 20 min (1000 rpm). It was then cooled in an ice bath, 150 μL of 96% ethanol was added and mixed vigorously at 2000 rpm. The samples were placed in a thermoshaker at 99 °C for 15 min (1000 rpm), then cooled and analyzed spectrophotometrically.

To the previously prepared samples, 1250 μL of demineralized water was added and mixed vigorously at 2000 rpm. 60 μL of the resulting solution was transferred to a new 2 mL Eppendorf tube to which 180 μL of water was added and then mixed vigorously at 2000 rpm. Then 20 μL of 80% aqueous phenol solution was added, followed by the rapid addition of 1200 μL of concentrated sulphuric acid using an automatic pipette (directing the acid stream directly into the centre of the solution layer). The sample was vortexed vigorously, allowed to stand for 30 min at room temperature, then transferred to a poly(methyl methacrylate) (PMMA) semi-micro cuvette and the absorbance measured at 490 nm. Measurements were performed triplicate, with pure water in the reference cuvette. Glycogen was quantified using an oyster glycogen standard (Sigma, G8751).

### 2.5 Fourier-transform infrared spectroscopy

Fourier-transform infrared (FTIR) spectroscopy technique was employed for the analysis of freeze-dried samples that were thoroughly homogenized prior to the measurement. Spectra were acquired using a Thermo Scientific Nicolet iS50 FTIR spectrometer (64 scans per spectrum, nominal resolution of 4 cm^−1^) quipped with a diamond attenuated total reflectance (ATR) accessory. The ATR-FTIR spectra were recorded in the 4000–400 cm^−1^ region.

### 2.6 Molecular weight determination

Molecular weight profiles of the glycogen-containing samples were determined by high-performance size exclusion chromatography (HP-SEC) (Tuvikene et al., 2015). The 0.05% sample in 0.1 M NaNO_3_ solution was prepared after solubilisation in a boiling water bath, filtered through a 0.22 μm RC membrane and 100 μL was injected into the HP-SEC system. The analysis was performed using a Shimadzu chromatograph (Kyoto, Japan) equipped with a DGU-20A5R degasser, Nexera X2 LC-30 CE pump, Nexera X2 SIL-30 AC autosampler, CTO-20 AC column oven, RID-10 A refractive index detector, Shodex OHpak SB-G (6.0 × 50 mm) guard column and two consecutive Shodex OHpak SB-806 M HQ (300 × 8 mm) columns (Tokyo, Japan) maintained at 60 °C. The mobile phase was 0.1 M NaNO_3_, flow rate 0.8 mL/min, analysis time 45 min. Pullulan standards (PSS GmbH, Germany) ranging from 0.342 to 2400 kDa were used to calibrate the system for determining the weight average molecular weights (Mw) of the samples by the LabSolutions software version 5.97 (Shimadzu, Kyoto, Japan).

### 2.7 Prebiotic effect determination

*Bifidobacterium animalis* subsp. *lactis* and *Cutibacterium acnes* subsp. *acnes* were grown in BSM and nutrient broth at 37 °C in anaerobic condition for 72 h. Microbial cells were seeded on 96-well plate in 50 μL amounts after adjusting the OD to 0.5, treated with 50 μL of mussel sample dissolved in water to obtain the final 0.5% concentration and incubated for 48 h at 37 °C. After 48 h, 10 μL of WST/ECS solution per well was added and the plate was incubated for 4 h in dark condition. Absorbance was measured at 460 nm by microplate reader FLUOstar OPTIMA (BMG LABTECH, San Diego, USA). Prebiotic effect was estimated as percentage change in viability relative to the control (50 μL water added instead of the sample) for the both microorganisms studied.

## 3 Results and discussion

### 3.1 Biomass characteristics and variations

On average, the dry matter content of the farmed mussels was 36%. Nearly 81% of the dry weight consisted of shell, while 19% was meat. The expectation that harvested mussels would exhibit significant seasonal variations in meat held true. The mean dry weight of mussels was significantly higher in summer compared to other seasons, and the proportion of meat in the dried biomass also peaked during this period. This coincides with the lowest ash content observed in both mussel meat and shells in summer (Table 2). Such variations in the dry matter content of mussel meat are well documented (Thompson and Bayne, 1974; Fernández et al., 2015; El Oudiani et al., 2016; Grkovic et al., 2023) and provide valuable insights into its potential applications. In particular, the meat-to-shell ratio, together with the average mass and dimensions of the mussels, play a crucial role in determining the yield and quality of biomass for commercial purposes. Higher average mussel mass and favorable meat-to-shell ratios indicate more efficient harvesting, with implications for both economic and nutritional value (Thompson and Bayne, 1974; Fernández et al., 2015; El Oudiani et al., 2016; Grkovic et al., 2023). In addition, meat colour varied seasonally, with darker meat observed in spring and lighter meat in autumn. This colour variability, possibly due to differences in pigment concentrations influenced by diet, environmental factors or genetics (Saraiva et al., 2011), could affect the visual appeal and perceived quality of the mussels, with implications for marketability (Saraiva et al., 2011). Ash content at different temperatures reveals significant information about the composition of the biomass. At 550 °C, organic material is burned off, leaving behind primarily inorganic constituents such as carbonates, which make up a major component of shell material. At 950 °C, the decomposition of carbonates and the release of carbon dioxide suggest the presence of significant carbonate content in the samples. The variation in ash content at two different temperatures could guide processing and utilization strategies for mussel and barnacle shells, possibly for mineral extraction or incorporation into other products. In addition, this information supports discussions on increasing the value of shellfish biomass, contributing to circular economy approaches in marine industries, as seen in literature focusing on the sustainable use of marine resources for food and nutraceutical applications (Granato et al., 2023; Lordan et al., 2011). On the other hand, the calcium carbonate from shells can be repurposed (Elegbede et al., 2023), complementing the nutritional analysis of the biomass itself as explored by Maar et al. (2023) and Kotta et al. (2020), who have highlighted the role of mussel farming in nutrient cycling and potential eutrophication control. The ability to extract value from all parts of the biomass, whether for nutritional content or mineral composition, aligns with global sustainability goals and the efficient use of natural resources.

**Table 2:** Characteristics of the mussel and barnacle biomasses used in the study. The sample abbreviations are M-1: Mechanically separated mussel meat collected in September 2020, M-2: Mechanically separated mussel meat collected in November 2021, M-3: Mechanically separated mussel meat collected in July 2022, M-4: Mechanically separated mussel meat collected in October 2022, B-2: Mechanically separated barnacle meat collected in November 2021, B-3: Mechanically separated barnacle meat collected in July 2022.

Sample	Average dry weight of one organism, g	Meat in dried biomass, %	Ash in dried meat, %		Ash in dried shells, %	
			550 °C	950 °C	550 °C	950 °C
M-1	0.30	13	7.6	5.7	93.6	53.5
M-2	0.27	16	5.6	5.1	93.8	53.8
M-3	0.39	30	5.6	5.0	92.9	53.6
M-4	0.27	16	9.4	6.9	94.0	53.7
B-2	0.08	10	19.7	15.0	92.9	54.1
B-3	0.13	12	18.0	14.6	92.3	54.1

### 3.2 Biomass processing

In this study, we employed a systematic and diverse fractionation strategy for blue mussel biomass, using different extraction, purification and fractionation techniques tailored to the characteristics of the biomass, including its small size and the inseparable but easily crushable nature of its soft shells (Fig. 1). This approach facilitated the isolation of bioactive and nutritionally relevant components such as proteins, peptides and glycogen.

The enzymatic treatment phase exploited the specificity of subtilisin to hydrolyze proteins into peptides and free amino acids, thereby enhancing the release of bioactive compounds. This step was essential not only for the inherent bioactivity of peptides and amino acids but also for the release of additional bioactive compounds from the biomass matrix (Gupta et al., 2002). Such enzymatic processes, by increasing protein digestibility and the solubility of the resulting peptides and amino acids, are critical for the production of bioavailable and functional nutraceutical ingredients (Daliri et al., 2018).

The processes of homogenization, centrifugation, drying, and alcohol precipitation were carefully controlled, taking into account the effect of heat treatment on ME-P and ME-P-H fractions. The variation in treatment conditions between these two fractions played a crucial role in elucidating the thermal stability, mobility, and bioactivity retention of different biomass components (Jin et al., 2016).

Ultrafiltration was employed to refine the fractions further, segregating compounds based on molecular weight, which is particularly relevant for the isolation of bioactive peptides with prebiotic properties. These compounds have been shown to have significant implications for gut health, as they can selectively stimulate the growth of beneficial gut flora (Gibson et al., 2017).

Glycogen extraction from blue mussel biomass used precise methods to maintain its structural integrity, essential for its role as an energy reserve and prebiotic potential (Ze et al., 2012). Controlled ethanol precipitation techniques preserved the molecular structure of glycogen, which is critical for its biological activity and fermentability by gut microbiota. This preservation ensures that intact glycogen molecules are preferentially metabolized by gut bacteria, facilitating the production of beneficial short-chain fatty acids (Morrison & Preston, 2016) highlighting the potential of glycogen in functional foods and nutraceuticals, supporting health through diet and sustainable use of marine resources.

The fractionation process developed in this study is adaptable, highlighted by the range of fractions produced, each with unique functional and prebiotic properties. Importantly, this method extends beyond mussels and is adaptable to various marine organisms, indicating the wider potential of marine biomasses as sources of bioactive compounds for diverse applications. The developed process outlines an optimized fractionation sequence tuned to the intrinsic properties of the biomass and the specifications of the target products. Future studies should investigate the bioactivity of peptides and amino acids in comparable marine biomasses, taking into account the effects of environmental conditions and seasonal variations on the composition and bioactivity of the derived fractions (El Oudiani et al., 2016; Thompson & Bayne, 2004; Grkovic et al., 2023; Fernández et al., 2015).

### 3.3 Protein content and amino acid profiles

**Fig. 2:**
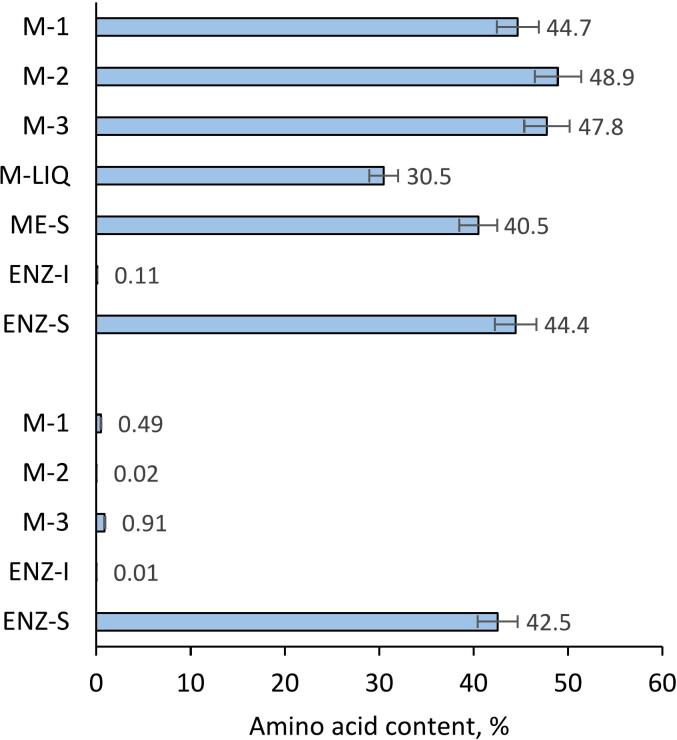
Total amino acid contents (top seven samples) and the quantities of free amino acids (bottom five samples) in the fractions obtained from the blue mussel biomass. Data shown as mean ± SD, *n* = 3. The sample abbreviations are M-1: Mechanically separated mussel meat collected in September 2020, M-2: Mechanically separated mussel meat collected in November 2021, M-3: Mechanically separated mussel meat collected in July 2022, M-LIQ: Substances from thawed water released from mussel biomass collected in July 2022, ME-S: Soluble part of homogenized mussel slurry collected in July 2022, ENZ-I: Insoluble part of subtilisin-treated mussel meat collected in July 2022, ENZ-S: Soluble part of subtilisin-treated mussel meat collected in July 2022. (For interpretation of the references to colour in this figure legend, the reader is referred to the web version of this article.)

Fig. 2 illustrates the amino acid profile of different fractions derived from blue mussel biomass, detailing both the total and free amino acid composition. No statistically significant differences in total amino acid content were observed in mechanically separated mussel flesh, with total amino acid levels approaching 50%, indicating a substantial presence of proteinaceous substances. Total amino acid concentrations were high in the thawed water of the mussel biomass and in the soluble fraction of the homogenized mussel slurry, but significantly lower in the separated mussel flesh. This suggests that mechanical separation and homogenization may affect amino acid retention. Notably, ENZ-S shows a comparable total amino acid content (44.4%) to the mechanically separated meat fractions, which aligns with previous research indicating that enzymatic treatment can preserve or even enhance the availability of amino acids in marine biomass preparations (Adler et al., 2022).

Analysis of free amino acid content showed that mussels harvested in summer have the highest levels, suggesting a higher initial presence of free amino acids in the biomass collected during this period. The expectation that enzymatic hydrolysis with subtilisin increases the extraction yield of essential amino acids from Baltic mussel biomass, thereby enhancing its prebiotic effects and nutritional value held true. The data for the ENZ-S fraction indicated an amino acid content of 42.5%, demonstrating the effectiveness of the enzymatic treatment in liberating amino acids into the solution. This high percentage suggests that subtilisin acted efficiently to break down proteins into amino acids that then dissolved in the surrounding medium. Unlike the minimal free amino acids observed in the ENZ-I fraction, the substantial total amino acids in ENZ-S reflect the enzyme's capacity to facilitate the transition of amino acids from bound to free states, which can be essential for enhanced bioavailability and potential bioactivity in subsequent applications. This aligns with the known efficiency of enzymatic hydrolysis in generating bioactive peptides and amino acids from protein-rich biomasses, offering valuable insights into the utility of such treatments in maximizing the nutritional yield from blue mussel biomass.

Comparing these results with previous studies reveals that the amino acid profiles of marine organisms are highly influenced by environmental factors, including diet, water temperature, and seasonal cycles, which can affect both the composition and total content of amino acids in the biomass (Cretton et al., 2023; Smaal & Haas, 1997). The observed variations in the free amino acid content across different fractions also underscore the complexity of enzymatic processes and their outcomes on the bioavailability of nutrients (Jin et al., 2016). Despite the enzymatic treatment, the ENZ-S fraction retains a high total amino acid content, indicating the effectiveness of enzymatic hydrolysis in releasing amino acids from protein complexes for subsequent utilization or analysis. This efficiency is important for the production of high quality nutraceuticals and functional foods where amino acid bioavailability is important (Gupta et al., 2002; Kim & Wijesekara, 2010).

**Fig. 3:**
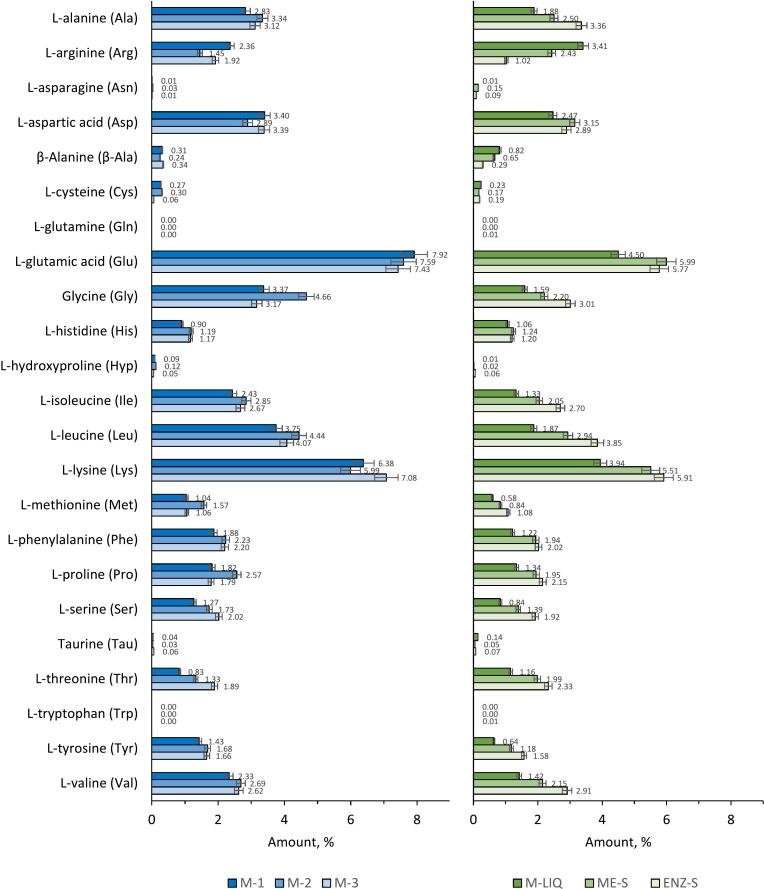
Amino acid profiles of the fractions obtained from the blue mussel biomass. SD < 5% for the analysis method. The sample abbreviations are M-1: Mechanically separated mussel meat collected in September 2020, M-2: Mechanically separated mussel meat collected in November 2021, M-3: Mechanically separated mussel meat collected in July 2022, M-LIQ: Substances from thawed water released from mussel biomass collected in July 2022, ME-S: Soluble part of homogenized mussel slurry collected in July 2022, ENZ-S: Soluble part of subtilisin-treated mussel meat collected in July 2022. (For interpretation of the references to colour in this figure legend, the reader is referred to the web version of this article.)

Amino acid analysis of blue mussel biomass did not show significant seasonal variations in the overall profiles of the mechanically separated fractions (M-1, M-2, M-3) (Fig. 3). The M-2 treatment showed the highest total amino acid content at 48.9%, while the M-3 treatment exhibited a notable lysine content of 7.08%. However, these variations were not sufficient to indicate a significant seasonal difference (*p* < 0.05). The consistency across different harvest times suggests that the amino acid composition of mussel meat is relatively stable regardless of season, underlining the potential reliability of mussel biomass as a source of essential amino acids for nutritional applications. This finding contrasts with the common assumption that significant environmental factors such as temperature and food availability can influence the nutritional composition of marine organisms between seasons. It highlights the robust nature of mussel meat composition, providing a reliable nutritional profile for consumers and processors alike. This analysis revealed a significant presence of glutamic acid in all fractions, with the soluble portion of the subtilisin-treated shellfish meat fraction containing 6.0%. This is consistent with observations in marine bivalve mollusks, which are known to contain high levels of glutamic acid, a critical component in protein synthesis (Dall & Moriarty, 1983). In addition, essential amino acids, including leucine and lysine, are prominent, highlighting their potential role in meeting nutritional requirements (Wu, 2009). The expectation that the valorization process of Baltic mussel biomass through specific biochemical techniques can significantly enhance its nutritional, health-promoting and commercial value held true. Directly extracted mussel meat fractions showed superior levels of these essential amino acids compared to those released from thawed mussel biomass and the soluble portion of homogenized mussel slurry. The variation in the amino acid content of different mussel fractions underlines the key influence of the processing techniques on the preservation and solubility of these nutrients, which is of crucial importance for their use in the food industry (Sikorski, 1990). This emphasizes the importance of optimizing processing methods to preserve essential amino acids, thereby enhancing the nutritional value of foods derived from the marine environment (Tacon et al., 2010).

### 3.4 Oil content and fatty acid profiles

**Fig. 4:**
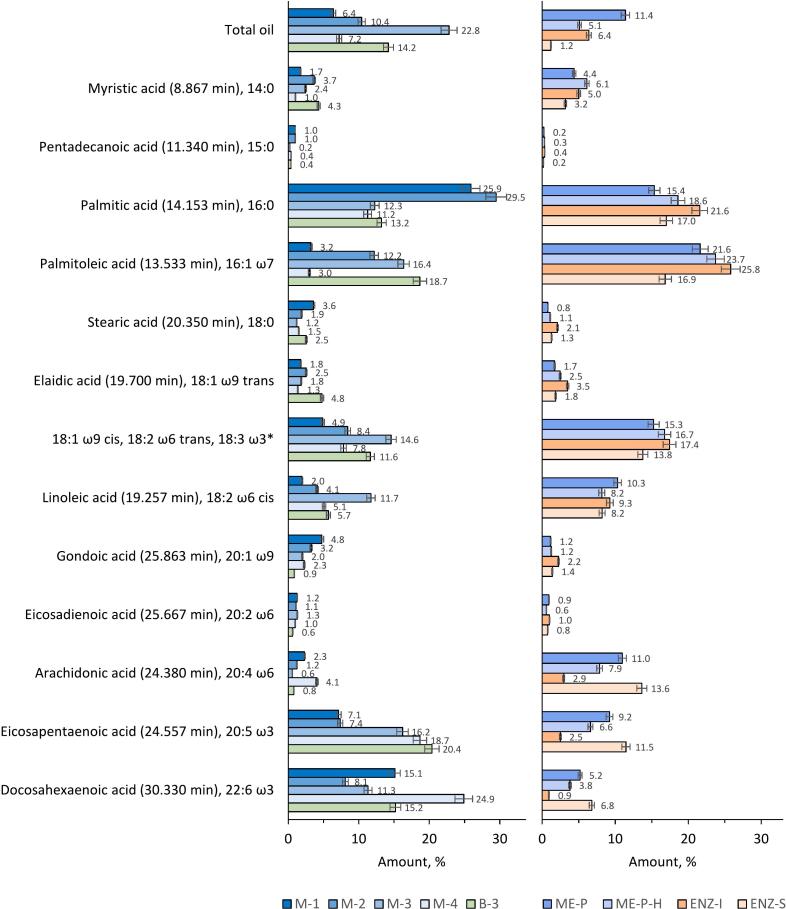
Fatty acid profiles of the fractions obtained from the blue mussel and barnacle biomasses. Values in brackets represent the GC retention times corresponding to the methyl ester derivatives of the respective fatty acids. * Sum of oleic acid (18:1 ω9 *cis*), linoelaidic acid (18:2 ω6 *trans*), α-linolenic acid (18:3 ω3), whose methyl derivatives at 19.493 min could not be separated by the used GC method. SD < 5% for the analysis method. The sample abbreviations are M-1: Mechanically separated mussel meat collected in September 2020, M-2: Mechanically separated mussel meat collected in November 2021, M-3: Mechanically separated mussel meat collected in July 2022, M-4: Mechanically separated mussel meat collected in October 2022, B-3: Mechanically separated barnacle meat collected in July 2022, ME-P: Purified mussel meat extract using a room-temperature process collected in July 2022, ME-P-H: Purified mussel meat extract using a high-temperature process collected in July 2022, ENZ-I: Insoluble part of subtilisin-treated mussel meat collected in July 2022, ENZ-S: Soluble part of subtilisin-treated mussel meat collected in July 2022. (For interpretation of the references to colour in this figure legend, the reader is referred to the web version of this article.)

The analytical data from mussel biomass revealed significant variation in oil content, with total oil percentages in the autumn harvests (M-2 and D-B-3) demonstrating notably higher values, peaking at 10.4% in M-2, as compared to the summer harvests (M-1 and D-M-4) where M-1 contained 6.4% total oil (Fig. 4). This finding illustrates the influence of seasonal variation and species-specific feeding behaviour on the accumulation of lipid reserves (Cretton et al., 2023; Ismail et al., 2016). The oil content data reported in this study are expressed relative to the dry weight of mussel meat, which presents a different context compared to many studies in literature that often report lipid content as a percentage of wet weight. When adjusted for moisture content, our findings show that the oil content in dried mussel meat fractions, with autumnal samples like M-2 reaching as high as 10.4%, could correspond to a lower percentage on a wet weight basis, potentially aligning with earlier studies. Lipid content in marine organisms such as *Mytilus edulis* typically ranges from 1% to 2% by wet weight, with variation dependent on environmental factors such as season and diet (Ackman, 1989; Smaal & Haas, 1997). This implies that when considering the water content in fresh mussel meat, our observed oil percentages in dry mass may parallel these established ranges, underlining the need for consistent measurement standards to enable accurate comparisons across studies. These findings highlight the potential of mussels and crustaceans as a viable alternative to traditional fish oils, especially when considering the fatty acid profiles identified in this study.

Focusing on the fatty acid profiles, Palmitic acid (16:0) predominated in autumn. This is in line with previous research identifying palmitic acid as a common fatty acid in marine species (Cretton et al., 2023). In addition, the fractions harvested in both autumn and summer had significant concentrations of polyunsaturated fatty acids (PUFAs), including particularly high levels of EPA and DHA. Such profiles highlight the nutritional importance of these fractions, particularly their potential contribution to anti-inflammatory and cardiovascular health benefits, paralleling the recognized benefits of EPA and DHA in human nutrition (Calder, 2015; Zanetti et al., 2015). Taking into account the similarity in oil content and fatty acid compositions between the mussel fractions and fish oil, it becomes evident that mussels, particularly those harvested in autumn and summer, could act as an equivalent source to the widely acclaimed fish oil, renowned for its rich ω-3 content. When considering barnacles in mussel biomass, it is important to be aware of the impact they can have on the composition of fatty acids. Barnacles have a unique lipid composition that could alter the fatty acid distribution in the biomass with a significant presence of these crustaceans, potentially increasing omega-6 fatty acid levels relative to the omega-3 rich profile characteristic of mussels. Consequently, samples with a high barnacle content may have an altered oil profile characterized by an increased ratio of omega-6 to omega-3 fatty acids.

Converting the dry weight oil content of mussel flesh to a wet weight basis allows more direct comparisons with the lipid content of typical shellfish and fish. Taking the highest dry weight oil content observed in our study, 10.4% for the M-2 fraction, and taking into account the usual water content of mussels, the recalculated oil content as a percentage of wet weight would fall within a lower range, potentially comparable to the 1–2% wet weight lipid content commonly reported for mussels, including in studies like those by Ackman (1989). This recalibrated value facilitates a better comparison with existing literature, where oily fish species are noted for their high oil content, which can reach 10–15% of the wet weight, not to mention the nearly pure oil content of fish oil supplements.

### 3.5 Glycogen separation and content

**Fig. 5:**
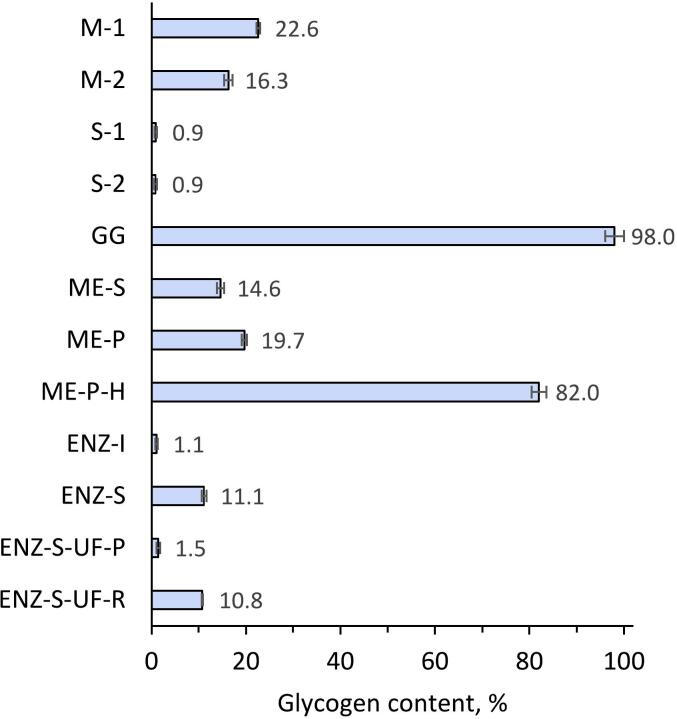
Glycogen contents of the fractions obtained from the blue mussel biomass. Data shown as mean ± SD, *n* = 3. The sample abbreviations are M-1: Mechanically separated mussel meat collected in September 2020, M-2: Mechanically separated mussel meat collected in November 2021, S-1: Mechanically separated mussel shells collected in September 2020, S-2: Mechanically separated mussel shells collected in November 2021, GG: Glycogen-rich fraction from mussel biomass collected in July 2022, ME-S: Soluble part of homogenized mussel slurry collected in July 2022, ME-P: Purified mussel meat extract using a room-temperature process collected in July 2022, ME-P-H: Purified mussel meat extract using a high-temperature process collected in July 2022, ENZ-I: Insoluble part of subtilisin-treated mussel meat collected in July 2022, ENZ-S: Soluble part of subtilisin-treated mussel meat collected in July 2022, ENZ-S-UF-P: Ultrafiltration permeate of soluble part of subtilisin-treated mussel meat collected in July 2022, ENZ-S-UF-R: Ultrafiltration retentate of soluble part of subtilisin-treated mussel meat collected in July 2022. (For interpretation of the references to colour in this figure legend, the reader is referred to the web version of this article.)

Seasonal fluctuations are a significant factor in determining the glycogen content in mussel biomass (Fig. 5). During colder months, when metabolic rates slow and food is scarcer, mussels tend to exhibit reduced glycogen levels as they utilize their stored energy. Contrarily, the levels generally increase when environmental conditions are less stressful and food is more abundant, usually in the warmer seasons. This cycle is evident in the current data, which shows a tendency for glycogen to decrease as the year progresses into winter, aligning with observations in similar marine organisms (Lemaire et al., 2006).

The current analysis of glycogen content reveals a notable variance across different fractions. The GG fraction is particularly rich in glycogen, with a content of 98%, suggesting the efficiency of the extraction process tailored for glycogen isolation. Meanwhile, the ME-P-H fraction, which has undergone heat treatment, displays a high glycogen content of 82%, indicating that controlled processing conditions can effectively preserve glycogen integrity and minimize thermal decomposition. These results underscore the impact of both environmental factors and processing methods on the glycogen content in mussel biomasses.

Contrastingly, the enzymatically treated fractions, ENZ-I and ENZ-S-UF-P, show markedly lower glycogen levels. Given that subtilisin targets proteins and not polysaccharides like glycogen, it is evident that the observed reduction in glycogen content is not a direct consequence of subtilisin action but may result from the separation processes post-enzymatic treatment. The ENZ-S fraction, with a glycogen content of 11.1%, and the ultrafiltration retentate, ENZ-S-UF-R, at 10.8%, further demonstrate the impact of molecular separation techniques on the distribution of glycogen.

The glycogen levels in the GG and ME-P-H fractions are substantially higher than those typically found in marine mollusks, which are reported to have glycogen contents ranging from 0.1% to 2% of dry weight (De Zwaan & Wijsman, 1976). The elevated glycogen levels achieved by the extraction and purification methods in this study underscore their efficacy and the potential of mussel-derived glycogen for high-energy applications, including sports and medical nutrition. These results highlight the significant effect of processing on glycogen yield and suggest the feasibility of developing glycogen-enriched products from mussel biomass for targeted nutritional and pharmaceutical applications.

### 3.6 Prebiotic effect of the fractions

**Fig. 6:**
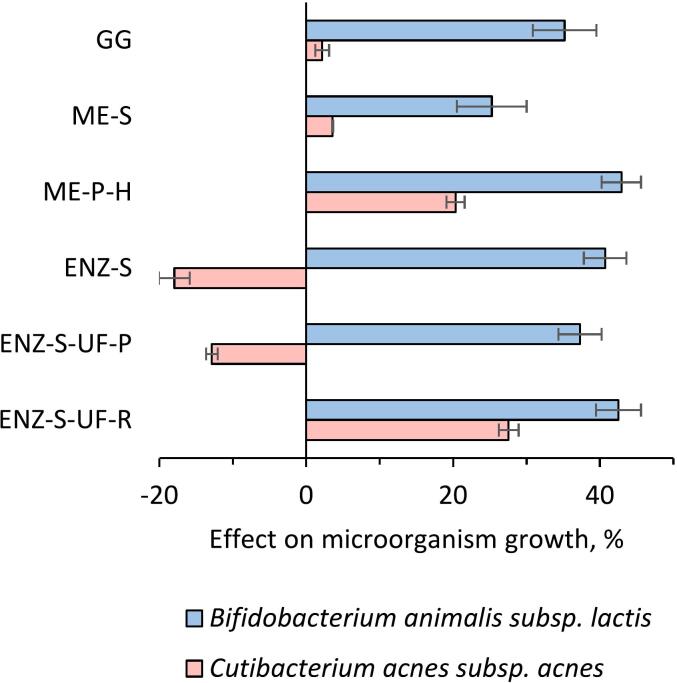
Effect of the fractions obtained from the blue mussel (at 0.5% concentration) on the growth of *Bifidobacterium animalis* subsp. *lactis* and *Cutibacterium acnes* subsp. *acnes*. Data shown as mean ± SD, *n* = 3. The sample abbreviations are GG: Glycogen-rich fraction from mussel biomass collected in July 2022, ME-S: Soluble part of homogenized mussel slurry collected in July 2022, ME-P-H: Purified mussel meat extract using a high-temperature process collected in July 2022, ENZ-S: Soluble part of subtilisin-treated mussel meat collected in July 2022, ENZ-S-UF-P: Ultrafiltration permeate of soluble part of subtilisin-treated mussel meat collected in July 2022, ENZ-S-UF-R: Ultrafiltration retentate of soluble part of subtilisin-treated mussel meat collected in July 2022. (For interpretation of the references to colour in this figure legend, the reader is referred to the web version of this article.)

Blue mussel-derived fractions show the potential prebiotic activity of *Bifidobacterium animalis* subsp. lactis (Fig. 6). The ME-P-H fraction, which was subject to high-temperature processing, notably promoted the growth of *B. animalis*. This suggests that certain processing conditions can preserve or enhance components such as prebiotic peptides and glycogen that benefit probiotic bacteria growth (Gibson & Roberfroid, 1995). However, regarding antimicrobial activity, none of the fractions in the presented data showed a negative impact on the growth of *C. acnes*. Instead, they all either had an insignificant or positive influence on bacterial growth.

Enzymatically treated fractions, especially the subtilisin-treated ENZ-S and ENZ-S-UF-P samples, demonstrated significant antimicrobial and prebiotic activity. This is likely due to their peptide size and solubility. Specifically, these samples reduced the growth of *C. acnes* by 12–18% and increased the survival of *B. animalis* by 37–40%, indicating the release of bioactive peptides following enzymatic processing (Corzo & Gilliland, 1999). These findings highlight the potential of mussel extract for applications in the food, pharmaceutical, and cosmeceutical industries. This enzymatic efficacy, however, appears to be contingent on molecular weight. The larger molecular weight fraction, ENZ-S-UF-R, did not demonstrate prebiotic activity, suggesting that smaller molecular weight peptides, often below 1 kDa, are preferentially utilized by probiotic bacteria and are more effective in prebiotic functions (Walsh et al., 2013).

**Fig. 7:**
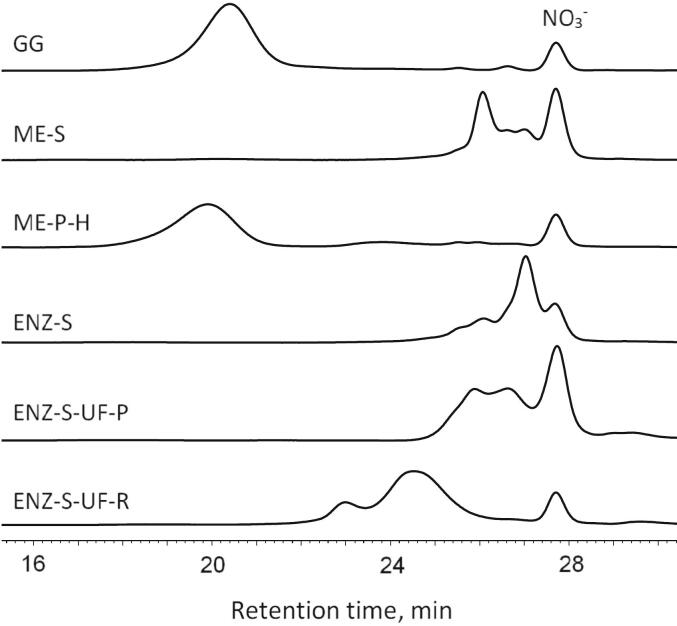
Size exclusion chromatography profiles of the fractions obtained from the blue mussel biomass. The nitrate peak designated by NO_3_^−^ is systemic and comes from the eluent used in the chromatography system. The sample abbreviations are GG: Glycogen-rich fraction from mussel biomass collected in July 2022, ME-S: Soluble part of homogenized mussel slurry collected in July 2022, ME-P-H: Purified mussel meat extract using a high-temperature process collected in July 2022, ENZ-S: Soluble part of subtilisin-treated mussel meat collected in July 2022, ENZ-S-UF-P: Ultrafiltration permeate of soluble part of subtilisin-treated mussel meat collected in July 2022, ENZ-S-UF-R: Ultrafiltration retentate of soluble part of subtilisin-treated mussel meat collected in July 2022. (For interpretation of the references to colour in this figure legend, the reader is referred to the web version of this article.)

Analysis showed that the GG fraction had a relatively uniform weight average molecular weight (Mw) distribution with an average of 310 kDa (Fig. 7). The Mw of ME-P-H was slightly higher at 416 kDa, the largest of the samples analyzed. The enzymatically treated samples contained predominantly components with Mw below 1 kDa, whereas ultrafiltration of these samples produced a retentate (ENZ-S-UF-R) with Mw of the primary component being approximately 5.4 kDa. These results indicate that preparations with Mw below 0.7 kDa have the most pronounced prebiotic effects. Prebiotic activity in preparations with the Mw below 0.7 kDa correlates with findings that probiotic bacteria use smaller peptides and oligosaccharides more efficiently, thereby enhancing their growth and activity (Gibson et al., 2017). This observation highlights the important role of molecular size in fermentation processes and suggests that lower molecular weight fractions may specifically influence the composition of the gut microbiota.

The Mw of glycogen exhibits considerable variability depending on its source and structural characteristics, with a wide range reported in the literature. For example, mammalian liver and muscle glycogen ranges from several hundred kDa to several thousand kDa (Roach et al., 2012). The documented Mw values of 310 kDa for the GG fraction and 416 kDa for the high-temperature processed mussel extract are at the lower end of this spectrum. This suggests that glycogen derived from blue mussel biomass may have a more branched structure or shorter chain lengths compared to mammalian glycogen.

For enzymatically treated preparations, the reported Mw below 1 kDa indicates the production of low molecular weight peptides. These findings are in line with studies demonstrating that enzymatic hydrolysis can effectively reduce protein size, resulting in the formation of oligopeptides and amino acids that can have various biological activities, including prebiotic effects (Daliri et al., 2018). The retentate from ultrafiltration showing the Mw around 5.4 kDa is consistent with the retention capabilities of ultrafiltration membranes, which are often used to separate peptides based on size (Toldrá et al., 2018).

The results of this study are consistent with the principle that the efficacy of prebiotics is determined by their physicochemical properties, which influence how they are fermented by the gut microbiota, resulting in health benefits (Gibson et al., 2017). Size exclusion chromatography clarifies this by showing that probiotic bacteria use lower molecular weight fractions more efficiently, resulting in enhanced prebiotic effects (Walsh et al., 2013). The results pave the way for the incorporation of mussel-derived fractions into functional foods and skin care products, exploiting their ability to selectively enhance beneficial microorganisms. This highlights the importance of marine-derived bioactives and their functional properties, opening up new opportunities for the development of health-promoting products.

### 3.7 Spectroscopic characteristics

**Fig. 8:**
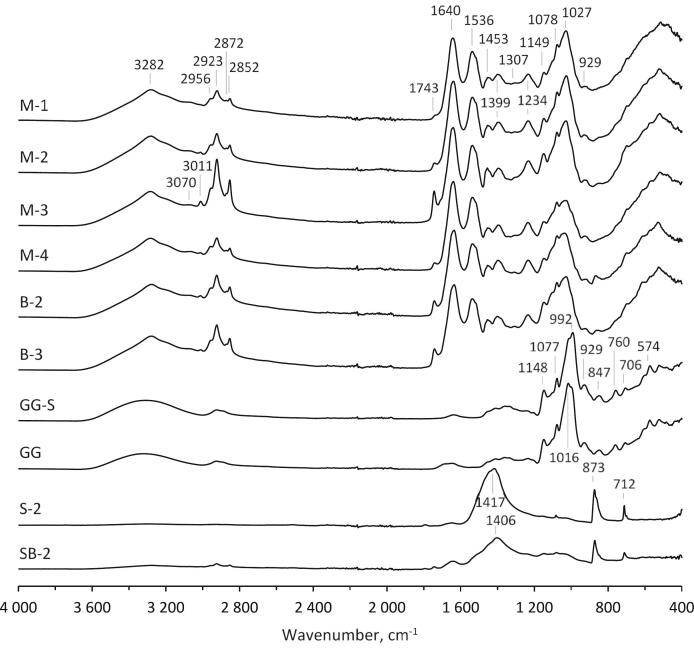
ATR-FTIR spectra of blue mussel meat (M-1, M-2, M-3, M-4), barnacle meat (B-2, B-3), glycogen from blue mussel (GG) and from oyster (GG-S, commercial sample from Sigma), and shells of blue mussel (S-2) and barnacle (SB-2). The sample abbreviations are M-1: Mechanically separated mussel meat collected in September 2020, M-2: Mechanically separated mussel meat collected in November 2021, M-3: Mechanically separated mussel meat collected in July 2022, M-4: Mechanically separated mussel meat collected in October 2022, B-2: Mechanically separated barnacle meat collected in November 2021, B-3: Mechanically separated barnacle meat collected in July 2022, GG-S: Glycogen-rich soluble fraction from mussel biomass collected in July 2022, GG: Glycogen-rich fraction from mussel biomass collected in July 2022, S-2: Mechanically separated mussel shells collected in November 2021, SB-2: Soluble part of barnacle homogenate collected in November 2021. (For interpretation of the references to colour in this figure legend, the reader is referred to the web version of this article.)

The ATR-FTIR spectra for the freeze-dried products/fractions derived from blue mussels (or barnacles) provide a valuable tool for rapid insight into their proximate chemical composition (Fig. 8). The meat fractions exhibit characteristic signals commonly associated with animal-derived samples rich in proteins, fatty acids, and carbohydrates.

Despite their complexity, the spectra allow differentiation of the main characteristic bands associated with proteins. Broad bands attributed to NH-stretching for amide A (∼3282 cm^−1^) and amide B (∼3070 cm^−1^), along with signals from CH_3_ symmetric stretching (2872 cm^−1^) and those from amide I (80% C=O stretching, 10% N—H bending, 10% C—N stretching) at 1640 cm^−1^, amide II (60% N—H bending, 40% C—N stretching) at 1536 cm^−1^ and amide III at 1307 cm^−1^, were observed, all predominantly arising from proteins (Bozkurt et al., 2012).

The olefinic =CH stretching vibration at 3011 cm^−1^ was assigned to unsaturated lipids and cholesterol esters (Zupančič et al., 2022). Other lipid-specific signals, including CH_2_ asymmetric stretching (2923 cm^−1^) and CH_2_ symmetric stretching (2852 cm^−1^), were also observed (Lozano et al., 2017). Furthermore, a distinctive band at 1743 cm^−1^, corresponding to saturated ester C=O stretching in phospholipids and cholesterol esters, was also present (Bozkurt et al., 2012). Signals characteristic of both lipids and proteins included CH_3_ asymmetric stretching at 2956 cm^−1^, CH_2_ bending at 1453 cm^−1^, and the band at 1399 cm^−1^ from fatty and amino acids due to COO^−^ symmetric stretching (Lozano et al., 2017). Additionally, the spectra of all meat samples exhibited bands from PO_2_^−^ asymmetric stretching (1234 cm^−1^) and symmetric stretching (1078 cm^−1^), inherent to phospholipids and nucleic acids (Zupančič et al., 2022).

Several carbohydrate-specific signals, such as a strong broad band at ∼1027 cm^−1^ from C—O bending in polysaccharides, and weaker vibrations at 1149 cm^−1^ (C—O—C asymmetric stretching) and 929 cm^−1^ (C—O—C stretching) of glycosidic linkages, were detected in the mussel and barnacle meat preparations, indicating the presence of glycogen (Bozkurt et al., 2012; Zupančič et al., 2022). The FTIR spectra of the glycogen isolated from blue mussel (sample GG) and the commercial glycogen sample from oyster were very similar, indicating similar purity, and revealed additional signals (847, 760, 706, 574 cm^−1^) in the fingerprint region associated with carbohydrates, not clearly observed for the meat samples.

The shells of mussel and barnacle exhibited three fundamental bands corresponding to C—O from carbonates: asymmetric stretching (1417–1406 cm^−1^), out-of-plane bending (873 cm^−1^) and in-plane bending (712 cm^−1^) (Ferraz et al., 2019). Additionally, several minor signals were observed in the spectrum of barnacle shells, suggesting the presence of residual meat particles. The finding is not unusual, as in the case of barnacles, the mechanical separation of meat and shells proved to be challenging. Contamination of meat by residues from shells could be detected by the sharp band at 873 cm^−1^, as this signal does not overlap with any signals from pure meat.

The consistent features in the FTIR spectra enable the rapid chemical profiling of blue mussel meat as well as that of barnacles, with the latter showing substantial similarities to those of mussels in their spectra. In such meats, the relative proportion of glycogen could be estimated based on the signal area in the range of 1186–877 cm^−1^, while lipids could be assessed using the integral of the bands at 2995–2800 cm^−1^. For lipids, this method demonstrated a very high linear correlation (R^2^ = 0.988) with oil contents measured gravimetrically. However, integration of the band in the region of 1774–1724 cm^−1^ arising from phospholipids and cholesterol esters, on the other hand, showed a slightly lower correlation (R^2^ = 0.965) with oil levels.

## 4 Conclusions

This study quantified the nutritional composition of mussel meat and demonstrated how strategic harvesting and processing can optimize nutrient profiles. The dry mass of mussel biomass varied, with meat content ranging from 13% to 30% and ash content ranging from 5.0% to 9.4% in dried meat. Amino acid analysis revealed the prominence of glutamic acid, leucine and lysine, up to 48.9% in certain fractions, while free amino acids were present in lower percentages. The oil content showed a remarkable range from 6.4% to 22.8%, with palmitic, myristic and oleic acids as the main fatty acids, and EPA and DHA levels varying significantly, indicating the health benefits of the mussels. Glycogen content was exceptionally high in some extracts, reaching up to 98%, illustrating the potential of these fractions for energy applications. In addition, enzymatically treated fractions showed significant prebiotic activity, supporting their potential use in various health-promoting applications. The results of the study on the bioactive compounds of blue mussel biomass offer new avenues for innovation in food and cosmetics, confirming the value of marine resources in advancing health and nutrition.

## CRediT authorship contribution statement

**Indrek Adler:** Writing – review & editing, Writing – original draft, Project administration, Data curation. **Jonne Kotta:** Writing – review & editing, Writing – original draft, Supervision. **Marju Robal:** Visualization, Investigation. **Sanjida Humayun:** Investigation, Formal analysis. **Kristel Vene:** Writing – review & editing, Validation. **Rando Tuvikene:** Writing – review & editing, Methodology, Conceptualization.

## Declaration of competing interest

The authors declare the following financial interests/personal relationships which may be considered as potential competing interests:

Rando Tuvikene reports financial support was provided by Estonian Research Council. Jonne Kotta reports financial support was provided by Horizon Europe. Jonne Kotta reports financial support was provided by European Maritime and Fisheries Fund. If there are other authors, they declare that they have no known competing financial interests or personal relationships that could have appeared to influence the work reported in this paper.

## Data Availability

Data will be made available on request.
